# Image quality and whole-lesion histogram and texture analysis of diffusion-weighted imaging of breast MRI based on advanced ZOOMit and simultaneous multislice readout-segmented echo-planar imaging

**DOI:** 10.3389/fonc.2022.913072

**Published:** 2022-08-12

**Authors:** Kun Sun, Hong Zhu, Bingqing Xia, Xinyue Li, Weimin Chai, Caixia Fu, Benkert Thomas, Wei Liu, Robert Grimm, Weiland Elisabeth, Fuhua Yan

**Affiliations:** ^1^ Department of Radiology, Ruijin Hospital, Shanghai Jiaotong University School of Medicine, Shanghai, China; ^2^ Department of Radiology, International Peace Maternity and Child Health Hospital, Shanghai Jiaotong University School of Medicine, Shanghai, China; ^3^ Department of Radiology, Ruijin Hospital Luwan Branch, Shanghai Jiaotong University School of Medicine, Shanghai, China; ^4^ MR Application Development, Siemens Shenzhen Magnetic Resonance Ltd., Shenzhen, China; ^5^ MR Application Predevelopment, Siemens Healthcare, Erlangen, Germany

**Keywords:** breast neoplasm, magnetic resonance imaging, diffusion weighted imaging, whole lesion, histogram analysis, texture analysis

## Abstract

**Objectives:**

To investigate the image quality and diagnostic capability a of whole-lesion histogram and texture analysis of advanced ZOOMit (A-ZOOMit) and simultaneous multislice readout-segmented echo-planar imaging (SMS-RS-EPI) to differentiate benign from malignant breast lesions.

**Study design:**

From February 2020 to October 2020, diffusion-weighted imaging (DWI) using SMS-RS-EPI and A-ZOOMit were performed on 167 patients. Three breast radiologists independently ranked the image datasets. The inter-/intracorrelation coefficients (ICCs) of mean image quality scores and lesion conspicuity scores were calculated between these three readers. Histogram and texture features were extracted from the apparent diffusion coefficient (ADC) maps, respectively, based on a WL analysis. Student’s t-tests, one-way ANOVAs, Mann–Whitney U tests, and receiver operating characteristic curves were used for statistical analysis.

**Results:**

The overall image quality scores and lesion conspicuity scores for A-ZOOMit and SMS-RS-EPI showed statistically significant differences (4.92 ± 0.27 *vs*. 3.92 ± 0.42 and 4.93 ± 0.29 *vs*. 3.87 ± 0.47, *p* < 0.0001). The ICCs for the image quality and lesion conspicuity scores had good agreements among the three readers (all ICCs >0.75). To differentiate benign and malignant breast lesions, the entropy of ADC_A-Zoomit_ had the highest area (0.78) under the ROC curve.

**Conclusions:**

A-ZOOMit achieved higher image quality and lesion conspicuity than SMS-RS-EPI. Entropy based on A-ZOOMit is recommended for differentiating benign from malignant breast lesions.

## Introduction

Breast MRI is the most sensitive imaging modality for the evaluation of breast cancer detection, diagnosis, and prognosis (1, 2). Diffusion-weighted imaging (DWI) can be used as an adjunct sequence to dynamic contrast-enhanced MRI (3–6).

Readout-segmented echo-planar imaging (RS-EPI) (7–9) with shortened echo spacing and echo train length (ETL), is associated with less geometric distortions and higher spatial resolution than single short echo-planar imaging (SS-EPI). However, the scan time of RS-EPI is significantly longer than that of SS-EPI. Simultaneous multislice excitation technique (SMS) allows one to acquire several slices in parallel so that fewer slice excitations are required to achieve the same slice coverage (10–12), thus improving the acquisition speed of RS-EPI DWI (13, 14).

The zoomed technique uses 2D radio-frequency pulses to excite a small field of view (FOV) in the phase-encoding direction thus shortening the readout ETL, with improved resolution, less geometric distortions, and less susceptibility artifact (15). The conventional zoomed technique is often associated with aliasing artifacts in the FOV due to the discretized sampling of the excitation k-space with resulting trajectory errors that lead to side excitations. Finsterbuch (16) proposed that a slight rotation of the field of excitation when performing zoomed EPI can mitigate potential aliasing artifacts, in the following referred to advanced ZOOMit (A-ZOOMit). Zoomed EPI DWI has been used on the prostate and other body regions (15, 17, 18). Equipped with complex-averaging and rigid motion registration among different b values and measurements, A-ZOOMit could achieve more excellent image quality and lesion conspicuity (18). Furthermore, no study has been published on breast tumors with the use of A-ZOOMit.

Whole-lesion (WL) histogram and texture analysis (19–21) show the probability distributions of continuous variables and the spatial distributions of gray values, which provides information about tumor heterogeneity. Previous studies (19, 22–24) show that histogram and texture analysis can achieve higher diagnostic accuracy compared with the use of only the mean values of parameters.

Hence, the objective of this study was to explore the image quality and feasibility of the A-ZOOMit and SMS-RS-EPI in clinical practice and to investigate the diagnostic capabilities of the WL histogram and texture analysis of A-ZOOMit and SMS-RS-EPI for further characterization of breast lesions.

## Materials and methods

### Study population

The local institutional review board approved this study. We obtained written informed consent from all participants. From February 2020 to October 2020, we enrolled 197 women with lesions suspicious for breast cancer on mammography or ultrasonography [i.e., Breast Imaging Reporting and Data System (BI-RADS) categories 4 or 5] who underwent SMS-RS-EPI and A-ZOOMit examinations. The exclusion criteria included the following: patients previously treated for a malignancy (n =10), patients without histopathological results (n = 5), patients with motion artifact (n =10), and patients with no lesion shown in DWI (n =5). For the 13 patients with multicentric or multifocal tumors, lesions with the largest sizes according to the postcontrast images were analyzed. Ultimately, 167 women (mean age, 53 years; age range, 22–82 years), with 167 lesions (mean size, 2.1 cm; range, 0.4–5.7 cm) were enrolled in the study.

### Magnetic resonance imaging scanning

All breast MRI examinations were performed on a 1.5-T system (MAGNETOM Aera; Siemens Healthcare, Erlangen, Germany) with a dedicated 18-channel phased-array breast coil. Before DCE-MRI, we performed axial bilateral fat-suppressed T2-weighted fast spin-echo imaging, prototype SMS-RS-EPI, and prototype A-ZOOMit on each patient. A-ZOOMit was performed with a slight rotation of the field of excitation, motion registration, and complex averaging, The other parameters of SMS-RS-EPI and A-ZOOMit are shown in **Table 1**. Apparent diffusion coefficient (ADC) maps were online-generated for the two DWI sequences.

**Table 1:** Sequence parameters for advanced ZOOMit (A-ZOOMit) and simultaneous multislice readout segmented echo-planar imaging (SMS-RS-EPI DWI).

Sequence Parameter	A-ZOOMit	SMS-RS-EPI
Diffusion mode	3D diagonal	3D diagonal
b values (s/mm^2^)	0,1,000	0,1,000
Average	b_0_ (7), b_1,000_ (21)	b_0_ (2), b_1,000_ (6)
Repetition time (ms)	5,700	3,780
Echo time	83	78
Orientation	Transversal	Transversal
FOV (mm^2^)	340*158	340*155
Scan matrix	220*102	220*100
Slice thickness (mm)	4	4
Slices	26	26
Readout segments	1	5
Oversampling in PE dir.	0	50%
No. of Sat.band	0	2
Fat suppression	SPAIR	SPAIR
Voxel size	1.5*1.5*4	1.5*1.5*4
Acquisition time	2:57	3:01
Bandwidth (Hz/Px)	988	668
Accel.factor PE	2	2
Accel factor slice	1	2
PE dir.	P ≥ A	P ≥ A

SMS-RS-EPI; simultaneous multislice (SMS) readout segmented echo-planar imaging; FOV, field of view; Px, pixel; PE, phase encoding. Sat. band, saturation band, which was used to suppress the signal from the back, to avoid the aliasing artifact; P, posterior; A, anterior; dir., direction.

### Multireader evaluation of image quality and lesion conspicuity scores

Three breast radiologists, each from a different hospital, independently evaluated and scored the overall image quality and lesion conspicuity of the SMS-RS-EPI and A-ZOOMit images (SK with 9 years of experience, XBQ with 6 years of experience, and LXY with 5 years of experience; **. performed the image quality measurement twice to calculate the intraclass agreement). All three radiologists were blinded to the sequence type when they evaluated the image quality and lesion conspicuity. The radiologists scored the overall image quality on a 5-point quality scale (1 = nondiagnostic, 2 = limited, 3 = diagnostic, 4 = good, and 5 = excellent). The radiologists scored lesion conspicuity on a scale of 1 (lesion not visible) to 5 (excellent visibility).

The signal-to-noise ratio (SNR) and contrast-to-noise ratio (CNR) of the lesions on DWI with b_1,000_ were also evaluated. SNR was defined as SNR = S_Lesion/_σ_noise_, where S_Lesion_ is the mean signal intensity of an region of interest (ROI) within the lesion, and σ_noise_ is the standard deviation (SD) of the background noise. CNR was defined as CNR = (S_Lesion –_ S_Tissue_)_/_σ_noise_, where S_Tissue_ is the mean signal intensity of an ROI on the normal breast tissue.

### Histogram and texture analysis based on apparent diffusion coefficient maps

Histogram and texture analyses for the SMS-RS-EPI- and A-ZOOMit-derived ADC maps were performed on the prototype MR Multiparametric Analysis software (Siemens Healthcare, Erlangen, Germany) by radiologists (**. and **) with four steps (19, 25). The four steps of data analysis were as follows:

1. Data loading: The ADC maps and b value of 1,000 images of both SMS-RESOLVE and A-ZOOMit were loaded to the software.
2. Seed point drawing: For ADC map analysis, foreground and background seed points were manually drawn inside and outside of the tumor on the three multiplanar reconstruction planes of the b_1,000_ images of SMS-RESOLVE.
3. Segmentation: The whole tumor was segmented by the software based on the seed points using a random walker algorithm. Manual adjustments were performed if the initial segmentation result was not satisfactory. The final three-dimensional (3D)-segmented volumes that were created on the b_1,000_ images were then automatically propagated to the A-ZOOMit maps.
4. Histogram and texture analyses: The WL histogram and texture analyses on the parametric maps were automatically performed by a one-push button. A total of seven histogram-based statistical features and four texture-based features were extracted. Histogram-based features included mean, SD, median, percentiles (5th and 95th), skewness (measure of asymmetry of the probability distribution), and kurtosis (measure of the shape of the probability distribution). Texture-based features included entropy (measure of the randomness of the gray levels), contrast (measure of the amount of gray-level variations), difference entropy (diff-entropy, measure of the entropy difference), and difference variance (diff-variance, measure of variation in the difference in gray levels between voxel pairs).

### Histopathologic analysis

The hematoxylin and eosin staining results and the immunohistochemical analysis of surgical specimens were reviewed in every patient’s medical record. Axillary lymph node metastasis; the expression status of Estrogen receptor (ER), Progesterone receptor (PR), and Human epidermal growth factor receptor-2 (HER2); and Ki-67 were routinely recorded.

### Statistical analysis

We described the clinical characteristics using frequencies for categorical

variables and means and ranges for all continuous variables. We compared the differences in clinical characteristics using chi-square tests and the analysis of variance. We used the Student’s t-test or one-way ANOVA in univariate analyses when the data were normally distributed and the Mann–Whitney U test when the data were not normally distributed.

We calculated the average scores from the three readers’ measurements and then used the Wilcoxon signed-rank tests to determine if differences existed between scores. We evaluated the intra- and interclass agreement of the readers’ scores. Then, we calculated the intra- and interclass agreement among the readers’ scores. The intraclass correlation coefficient (ICC_intra_) was computed from radiologist 1’s two measurements. The interclass correlation coefficients were computed between radiologist 1’s first measurements and radiologist 2’s and radiologist 3’s measurements (ICC_1,2_ and ICC_1,3_, respectively). We interpreted an ICC greater than 0.75 as indicative of good agreement.

We used SPSS (v. 26.0; SPSS, Chicago, IL, USA) and MedCalc (MedCalc, Mariakerke, Belgium) for the statistical analyses. We considered a *p*-value less than 0.05 indicative of statistically significant difference.

## Results

### Clinical characteristics

There were significant differences in demographic characteristics between patients with malignant lesions (mean age, 55.0 ± 11.7 years; range, 28–81 years) and patients with benign lesions (mean age, 48.5 ± 11.7 years; range, 22–82 years; *p* = 0.001).

### Pathological features

Of the 167 lesions, 110 were malignant, and 57 were benign. There was a significant difference in the lesion size between malignant and benign breast lesions (2.27 ± 0.94 cm *vs*. 1.71 ± 0.98 cm, *p* < 0.0001).

The malignant lesions included ductal carcinoma *in situ* (N = 18), invasive carcinoma of no special type (N = 87), invasive lobular carcinoma (N = 1), invasive solid papillary carcinoma (N = 1), mucinous carcinoma (N = 2), and encapsulated papillary carcinoma with invasion (N = 1). Of the 92 invasive breast cancers in this study, 21 (22.8%) were luminal A cancer, 30 (32.6%) were luminal B cancer, 33 (35.9%) were HER2-positive cancer, and 8 (8.7%) were triple-negative cancer. Of all these invasive cancers, there were 61 (66%) patients who were lymph node–negative, and there were 31 (34%) patients who were lymph node–positive.

Benign lesions included fibroadenoma (N = 24), benign phyllodes tumors (N = 1), fibrocystic change (N = 7), cyst-combined chronic infection (N = 9), papilloma (N = 8), usual ductal hyperplasia (N = 4), and adenosis (N = 4).

### Image quality score comparisons of SMS-RS-EPI and A-ZOOMit

The mean overall image quality scores of A-ZOOMit and SMS-RS-EPI showed a statistically significant difference (4.92 ± 0.27 *vs*. 3.92 ± 0.42, *p* < 0.0001, respectively, in the multireader study).

**Figure 1:**
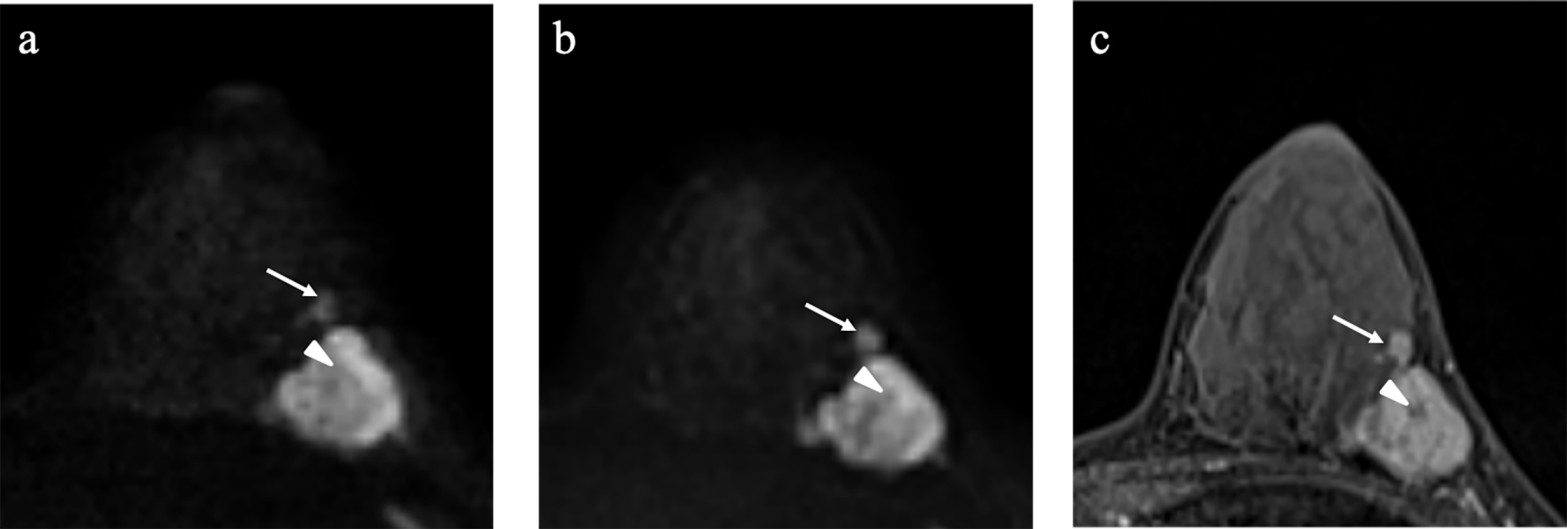
Example images of a 35-year-old woman with invasive ductal carcinoma in the left breast **(A-C)**. SMS-RS-EPI image of b_1,000_ **(A)**; advanced ZOOMit (A-ZOOMit) image of b_1,000_ **(B)**; dynamic contrast imaging of T1WI **(C)**. A-ZOOMit image showed a better image quality of the satellite nodule (long arrow) and necrosis (short arrow) than the SMS-RS-EPI image.

For the image quality score of A-ZOOMit images, the ICC_intra_ was 0.94, ICC_1,2_ was 0.79, ICC_2,3_ was 0.80, and ICC_1,3_ was 0.85. For the SMS-RS-EPI, the ICC_intra_ was 0.92, ICC_1,2_ was 0.85, ICC_2,3_ was 0.77, and ICC_1,3_ was 0.75. The details of ICCs are shown in **Table 2**. A case of b_1,000_ based on SMS-RESOLVE and A-ZOOMit is shown in **Figure 1**.

**Table 2:** Intra- and interclass correlation coefficients of multireader ratings of image-quality and lesion conspicuity on A-ZOOMit and SMS-RS-EPI.

	Radiologist 1	Radiologist 3
***A-ZOOMit***		
Image Quality		
Radiologist 1	0.94 (0.84–0.91)	0.75 (0.50–0.99)
Radiologist 2	0.79 (0.60–0.97)	0.83 (0.68–0.98)
Lesion Conspicuity		
Radiologist 1	0.90 (0.87–0.93)	0.80 (0.71–0.89)
Radiologist 2	0.83 (0.71–0.96)	0.80 (0.70–0.90)
***SMS-RS-EPI***		
Image Quality		
Radiologist 1	0.92 (0.90–0.94)	0.78 (0.66–0.89)
Radiologist 2	0.85 (0.69–1.0)	0.77 (0.63–0.91)
Lesion Conspicuity		
Radiologist 1	0.86 (0.82–0.89)	0.80 (0.71–0.89)
Radiologist 2	0.80 (0.69–0.90)	0.81 (0.70–0.92)

Data in parentheses represent the 95% confidence interval. SMS-RS-EPI; simultaneous multislice (SMS) readout-segmented echo-planar imaging.

### Lesion conspicuity score comparisons of SMS-RS-EPI and A-ZOOMit

The mean lesion conspicuity scores of the A-ZOOMit and SMS-RS-EPI showed a significant difference (4.93 ± 0.29 *vs*. 3.87 ± 0.47, *p* < 0.0001, respectively, in the multireader study).

For the lesion conspicuity score of A-ZOOMit images, the ICC_intra_ was 0.94, the ICC_1,2_ was 0.77, the ICC_2,3_ was 0.83, and the ICC_1,3_ was 0.75. For the SMS-RS-EPI, the ICC_intra_ was 0.90, the ICC_1,2_ was 0.83, the ICC_2,3_ was 0.81, and the ICC_1,3_ was 0.78.

### Histogram and texture analyses of ADC in differentiating malignant and benign breast lesions

**Figure 2:**
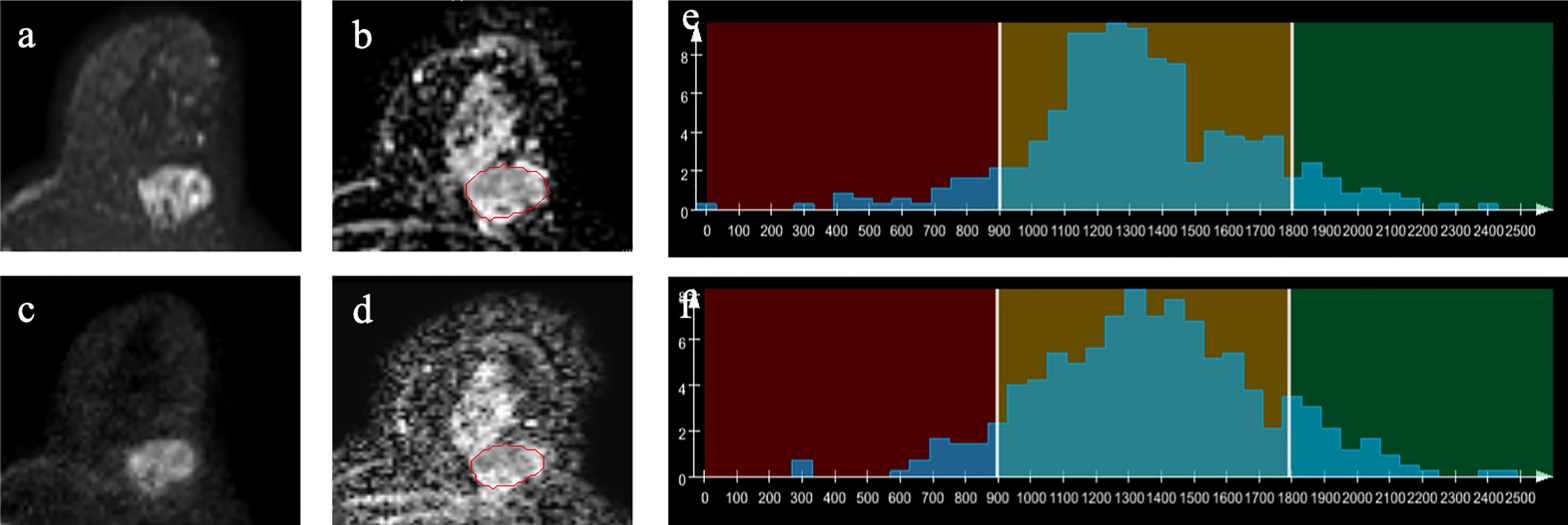
Example images of a 60-year-old woman with invasive ductal carcinoma in the left breast **(A–D)**. A-ZOOMit image of b_1,000_ **(A)**; apparent diffusion coefficient (ADC) map based on A-ZOOMit **(B)**; SMS-RS-EPI image of b_1,000_ **(C)**; ADC map based on SMS-RS-EPI **(D)**; histogram of segmented tumors based on ADC maps **(E, F)**.

**Figure 3:**
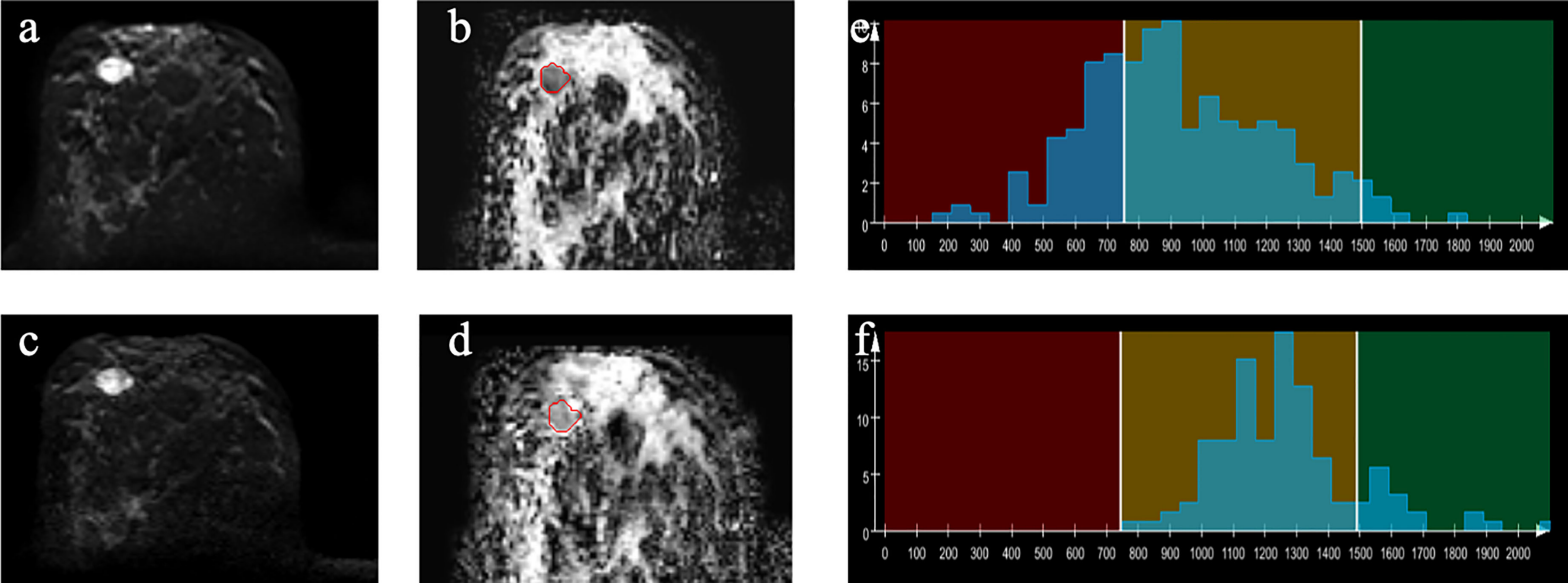
Example images of a 44-year-old woman with papilloma in the right breast **(A–D)**. A-ZOOMit image of b_1,000_ **_(_a_)_**; ADC map based on A-ZOOMit **(B)**; SMS-RS-EPI image of b_1,000_ **(C)**; ADC map based on SMS-RS-EPI **(D)**; histogram of segmented tumors based on ADC maps **(E, F)**.

The histogram and texture features to distinguish between malignant and benign breast lesions are shown in **Table 3** and **Supplementary Table 1**. Imaging examples are shown in **Figures 2**, **3**.

**Table 3:** Histogram and texture analysis of apparent diffusion coefficient values based on SMS-RS-EPI and A-ZOOMit between malignant and benign breast lesions.

Characteristics	Benign Lesions	Malignant Lesions	*p*-values
***A-ZOOMit***			
Mean	1.29 ± 0.28	1.14 ± 0.23	<;0.0001*
Median	1.30 ± 0.29	1.12 ± 0.25	<;0.0001*
5th percentile	0.63 ± 0.31	0.52 ± 0.21	0.011
Skewness	-0.18 ± 0.54	0.21 ± 0.56	<;0.0001*
Diff-entropy	2.11± 0.18	2.27 ± 0.19	<;0.0001*
Entropy	3.09 ± 0.21	3.27± 0.19	<;0.0001*
***SMS-RS-EPI***			
Mean	1.18 ± 0.28	1.07± 0.22	0.008
Median	1.20 ± 0.32	1.05 ± 0.24	0.001*
Skewness	-0.26 ± 0.57	0.20 ± 0.51	<;0.0001*
Diff-entropy	2.15 ± 0.19	2.25 ± 0.13	<;0.0001*
Entropy	3.13 ± 0.25	3.29 ± 0.12	<;0.0001*

SMS-RS-EPI; simultaneous multislice (SMS) readout-segmented echo-planar imaging; SD, standard deviation. * symbol represent significant difference.

The mean, median, and 5th percentile of the ADCs based on A-ZOOMit were significantly lower in the malignant lesions than in the benign tumors (*p* < 0.0001, < 0.0001, 0.011, respectively). However, the skewness, entropy, and diff-entropy of the ADCs based on A-ZOOMit were significantly higher in the malignant lesions than in the benign tumors (*p* < 0.0001, < 0.0001, < 0.0001, respectively).

The mean and median value of the ADCs based on SMS-RS-EPI were significantly lower in the malignant lesions than in the benign tumors (*p* = 0.008, 0.001, respectively). However, the skewness, entropy, and diff-entropy of the ADCs based on SMS-RS-EPI were significantly higher in the malignant lesions than in the benign tumors (*p* < 0.0001, < 0.0001, < 0.0001, respectively).

### Differences of SNR and CNR between SMS-RS-EPI and A-ZOOMit

**Figure 4:**
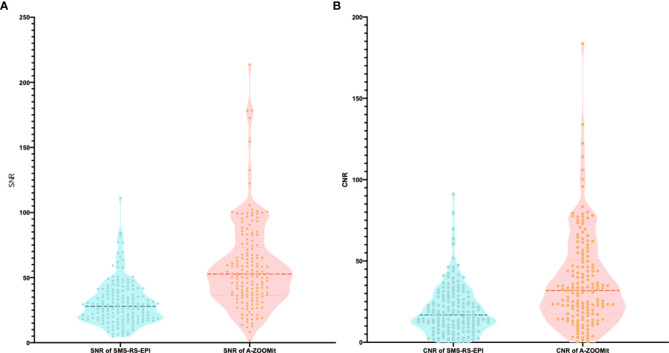
The SNR and CNR of b1,000 based on SMS-RS-EPI and A-ZOOMit. **(A, B)** There was a significant difference of SNR of b1000 based on SMS-RESOLVE and A-ZOOMit (p < 0.001) **(A)**; There was a significant difference of SNR of b1000 based on SMS RESOLVE and A-ZOOMit (p < 0.001) **(B)**.

There was a significant difference of the SNR_b1,000_ between the SMS-RS-EPI and A-ZOOMit images (30.62 ± 16.95 *vs*. 58.19 ± 33.34, *p* < 0.0001). Furthermore, the CNR_b1,000_ based on SMS-RS-EPI and A-ZOOMit also shown significant difference (19.99 ± 15.10 *vs*. 37.42 ± 28.12, *p* < 0.0001 **Figure 4**).

### Results of the receiver operating characteristic curves

**Figure 5:**
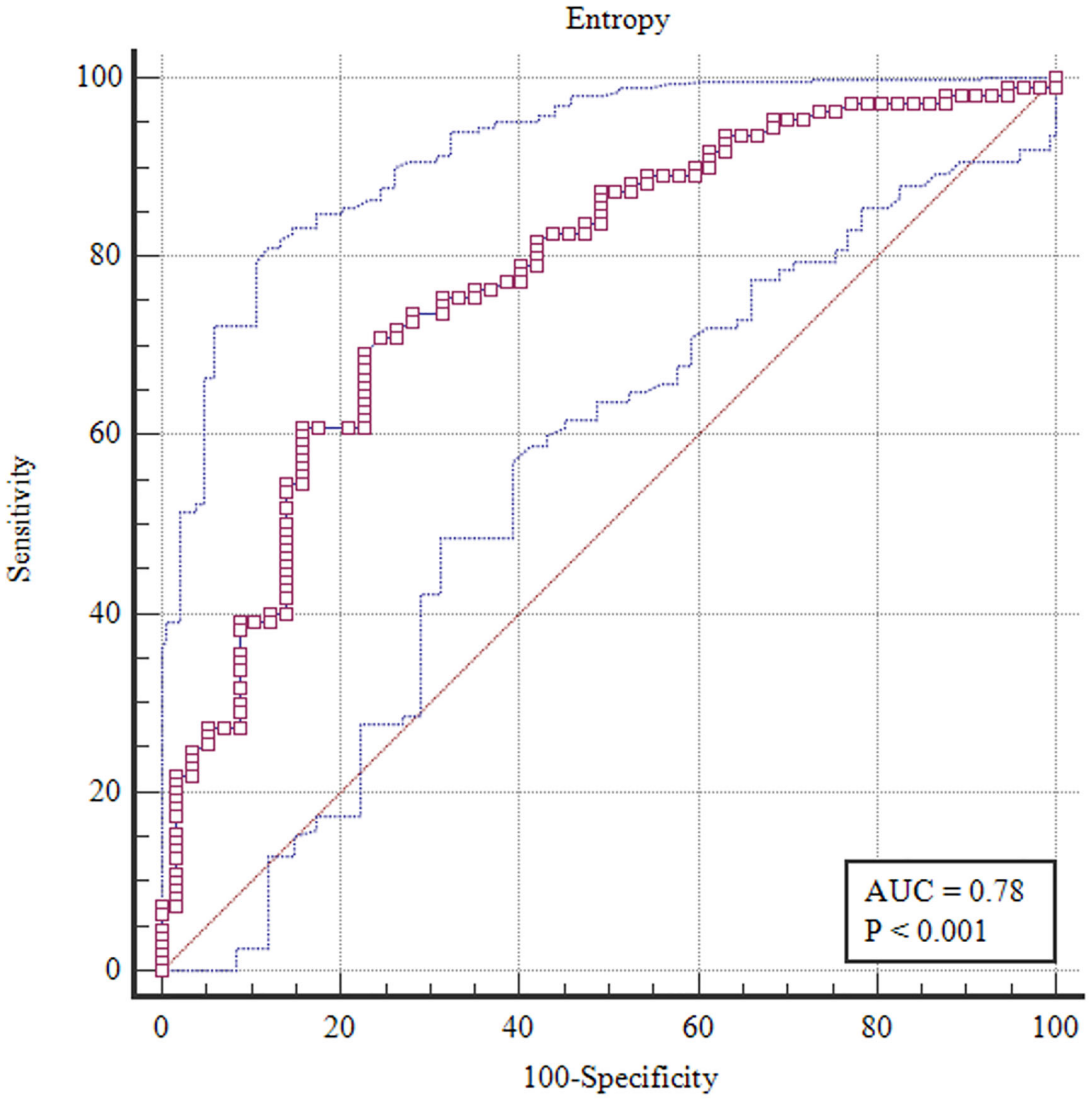
Receiver operating characteristic curve of entropy with 95% confidence interval based on A-ZOOMit for the differentiation between benign and malignant lesions.

To differentiate the benign from malignant breast lesions, the entropy value of ADC (0.78, 95% CI 0.71–0.84) from the A-ZOOMit texture analysis had the highest area under the ROC curve (**Figure 5**). The details of areas under the ROC curves are shown in **Supplementary Table 2**.

## Discussion

In our study, we found a significantly higher image quality and lesion conspicuity of breast DWI based on A-ZOOMit, with an improved diagnostic performance of texture parameter entropy for differentiating between malignant and benign breast lesions, compared with SMS-RS-EPI.

Previous studies (15, 17, 18) using zoomed EPI were based on the conventional ZOOMit method. In our study, we used advanced ZOOMit, which could be able to further improve the image quality and the accuracy of the ADC estimation. SMS-RS-EPI has already been used in breast cancer diagnosis (14, 26, 27). Hu et al. (27) found that SMS-RS-EPI can significantly reduce the acquisition time and achieve a comparable diagnostic accuracy for the differentiation between malignant and benign breast lesions. In our study, we used identical scan time and the same b values for both A-ZOOMit and SMS-RS-EPI. We wanted to know which sequence would be the most preferred DWI sequence for the breast radiologists on a 1.5-T MRI scanner.

Our results showed that both the image quality and lesion conspicuity based on A-ZOOMit were higher than with SMS-RS-EPI in multireader studies. Furthermore, the appearance of the tumor details shown on A-ZOOMit were more suitable for radiologists’ reading habits. The quantitative evaluation demonstrated that the SNR and CNR of the lesion on A-ZOOMit were also higher than that of SMS-RS-EPI. The better image quality, lesion conspicuity, and higher SNR and CNR of A-ZOOMit can be attributed to the higher number of averaging, the complex averaging scheme, and motion registration. As the zoomed FOV technique was used, neither oversampling in phase-encoding direction nor the saturation band on the back region were needed to avoid the aliasing artifacts for A-ZOOMit; thus, the scan time can be saved, and a higher number of averaging can be used compared to SMS-RS-EPI. Considering the benefits mentioned above, we recommended A-ZOOMit DWI for clinical breast application on a 1.5-T MRI scanner.

Our results show that most of the histogram and texture features of ADC can be used for the diagnosis of breast cancer. Suo et al. (28) also found that the entropy of ADC provided complementary information for evaluating IDC phenotypes. Additionally, in our study, the entropy of ADC based on A-ZOOMit showed the highest area under the ROC curves for the diagnosis of breast cancer, which was consistent with their study. Higher entropy represents higher cellular heterogeneity. Cellular heterogeneity among breast cancers may correlate with the histopathological changes of the hormone receptor status and HER2 status (29, 30). In our study, the HER2-positive cancer counts for 35.9%, which will be shown as a higher entropy value.

Our study had several limitations. First of all, the limited sample size and the imbalanced distribution of benign lesions. Second, we generated and analyzed only 11 commonly used texture features proved valuable in previous clinical applications (31). In addition, anisotropic voxel resolutions in our DWI data may not allow fully appreciating the 3D textural structure of the lesions. In our further study, we will enroll more high-order features to reflect tumor heterogeneity. Third, we used the background noise to estimate the noise level of the lesions for the calculation of SNR and CNR since the individual images were not available. This method is not suitable for estimating the noise when parallel imaging acceleration is used. In our future studies, we will utilize the method described by Reeder SB et al. (32). Fourth, we compared the two newly developed DWI sequences to each other and did not compare them with conventional SS-EPI DWI and/or RS-EPI sequences since many studies (7, 14, 33) have already compared them. Further large-scale multicenter studies could provide an evidence of the effect of advanced DWI methods on diagnostic accuracy.

We concluded that DWI based on A-ZOOMit provides significantly higher image quality and lesion conspicuity than SMS-RS-EPI in our study. Thereby, texture analysis based on A-ZOOMit achieved higher diagnostic accuracy for the differentiation of benign and malignant breast lesions.

## Data availability statement

The raw data supporting the conclusions of this article will be made available by the authors, without undue reservation.

## Ethics statement

This study was reviewed and approved by Ruijin Hospital, Shanghai Jiaotong University School of Medicine. The patients/participants provided their written informed consent to participate in this study.

## Author contributions

Literature search: KS, and HZ. Study design: KS, and FY. Data collection: KS, HZ, and WC. Data analysis: KS, BX and XL. Manuscript editing: WC, CF, BT, WL, RG, and WE. Manuscript review: KS and FY. All authors contributed to the article and approved the submitted version.

## Funding

This work was supported by the National Natural Science Foundation of China (No. 81801651).

## Conflict of interest

Authors CF and WL were employed by Siemens Shenzhen Magnetic Resonance Ltd. Authors BT, RG and WE were employed by Siemens Germany Magnetic Resonance Ltd.

The remaining authors declare that the research was conducted in the absence of any commercial or financial relationships that could be construed as a potential conflict of interest.

## Publisher’s note

All claims expressed in this article are solely those of the authors and do not necessarily represent those of their affiliated organizations, or those of the publisher, the editors and the reviewers. Any product that may be evaluated in this article, or claim that may be made by its manufacturer, is not guaranteed or endorsed by the publisher.

## Abbreviations

AUC, area under the receiver operating characteristic curve; BI-RADS, breast imaging reporting and data system; ROC, receiver operating characteristic; WL, whole lesion; DWI, diffusion-weighted imaging; SMS, simultaneous multislice; A-ZOOMit, advanced ZOOMit; RS-EPI, readout segmented echo-planar imaging; SS-EPI, single-shot echo-planar imaging; EPI, echo-planar imaging; ETL, echo train length; ADC, apparent diffusion coefficient; FOV, field of view; ICCs, inter-/intracorrelation coefficients; CI, confidence interval; SNR, signal-to-noise ratio; CNR, contrast-to-noise ratio. ; MRI, magnetic resonance imaging; ROI, region of interest; ER, Estrogen receptor; PR, Progesterone receptor; HER2, Human epidermal growth factor receptor-2.
