# Supplementary material for: Image quality and whole-lesion histogram and texture analysis of diffusion-weighted imaging of breast MRI based on advanced ZOOMit and simultaneous multislice readout-segmented echo-planar imaging
Source: Front Oncol. 2022 Aug 12;12:913072. doi: 10.3389/fonc.2022.913072 (PMC9411810; doi:10.3389/fonc.2022.913072)
Supplement: Supplementary file 1 [file Table_1.docx]

Supplementary Material

Supplementary Table 1

| **Characteristics** | **Benign Lesions** | **Malignant Lesions** | ***p* values** |
| --- | --- | --- | --- |
| ***A-ZOOMit*** |  |  |  |
| SD | 0.39 ± 0.13 | 0.40 ± 0.08 | 0.658 |
| 95th percentile | 1.90 ± 0.33 | 1.83 ± 0.28 | 0.136 |
| Kurtosis | 0.09 ± 0.96 | 0.39 ± 0.95 | 0.054 |
| Diff-variance | 4.66 ± 1.91 | 5.17 ± 2.00 | 0.112 |
| ***SMS-RS-EPI*** |  |  |  |
| SD | 0.39 ± 0.13 | 0.38 ± 0.08 | 0.645 |
| 5th percentile | 0.52 ± 0.30 | 0.47 ± 0.25 | 0.231 |
| 95th percentile | 1.79 ± 0.38 | 1.74 ± 0.26 | 0.269 |
| Kurtosis | 0.37 ± 1.20 | 0.39 ± 0.88 | 0.923 |
| Diff-variance | 5.43 ± 2.58 | 5.08 ± 1.81 | 0.308 |
| Contrast | 20.11 ± 8.37 | 19.26 ± 6.13 | 0.454 |

Supplementary Table 2

ROC analysis of ADC values based on SMS-RS-EPI and A-ZOOMit between malignant and benign breast lesions

| **Characteristics** | **AUCs** | **95% CI** |
| --- | --- | --- |
| ***A-ZOOMit*** |  |  |
| Mean | 0.67 | 0.58 - 0.76 |
| Median | 0.70 | 0.62 - 0.79 |
| 5% | 0.60 | 0.49 - 0.69 |
| Skewness | 0.70 | 0.62 - 0.78 |
| Entropy | 0.78 | 0.70 - 0.85 |
| Diff-entropy | 0.75 | 0.67 - 0.83 |
| ***SMS-RS-EPI*** |  |  |
| Mean | 0.64 | 0.54 - 0.73 |
| Median | 0.68 | 0.59 - 0.77 |
| Skewness | 0.72 | 0.64 - 0.81 |
| Entropy | 0.71 | 0.62 - 0.79 |
| Diff-entropy | 0.66 | 0.57 - 0.76 |

AUCs represents areas under the receiver operating characteristic (ROC) curve; CI, confidence interval; SMS-RS-EPI; simultaneous multi-slice (SMS) readout segmented echo-planar imaging
